# ATD: a comprehensive bioinformatics resource for deciphering the association of autophagy and diseases

**DOI:** 10.1093/database/bay093

**Published:** 2018-09-18

**Authors:** Wenjing Wang, Peng Zhang, Leijie Li, Zhaobin Chen, Weiyang Bai, Guiyou Liu, Liangcai Zhang, Haiyang Jia, Li Li, Yingcui Yu, Mingzhi Liao

**Affiliations:** 1 College of Life Sciences, Northwest A&F University, Yangling, Shaanxi, China; 2 School of Medicine, Tongji University, Shanghai, China; 3 School of Life Science and Technology, Harbin Institute of Technology, Harbin, China; 4 Department of Statistics, Rice University, Houston, TX, USA; 5 College of Computer Science and Technology, Key Laboratory of Symbolic Computation and Knowledge Engineering of Ministry of Education, Jilin University, Changchun, China; 6 College of Natural Resources and Environment, Northwest A&F University, Yangling, Shaanxi, China

## Abstract

Autophagy is the natural, regulated, destructive mechanism of the eukaryotes cell that disassembles unnecessary or dysfunctional components. In recent years, the association between autophagy and diseases has attracted more and more attention, but our understanding of the molecular mechanism about the association in the system perspective is limited and ambiguous. Hence, we developed the comprehensive bioinformatics resource Autophagy To Disease (ATD, http://auto2disease.nwsuaflmz.com) to archive autophagy-associated diseases. This resource provides bioinformatics annotation system about genes and chemicals about autophagy and human diseases by extracting results from previous studies with text mining technology. Based on the big data from ATD, we found that some classes of disease tend to be related with autophagy, including respiratory disease, cancer, urogenital disease and digestive system disease. We also found that some classes of autophagy-related diseases have a strong association among each other and constitute modules. Furthermore, we extracted the autophagy–disease-related genes (ADGs) from ATD and provided a novel algorithm Optimized Random Forest with Label model to predict potential ADGs. This bioinformatics annotation system about autophagy and human diseases may provide a basic resource for the further detection of the molecular mechanisms of autophagy pathway to disease.

## Introduction

Autophagy is a process of self-digestion that generally sequesters cytoplasmic components in double-membrane vesicles and degrades them by regulating the function of lysosome (1). Autophagy promotes the orderly degradation and recycling of cellular components (2). This process is known as an adaptation of eukaryotes against negative milieus (3, 4). Previous studies show that impaired function of autophagy leads to the pathogenesis of some human diseases like cancer and neurodegenerative diseases (5, 6). Some studies reported that autophagy in cancer cells is induced as a mechanism to promote their survival (7, 8). On the other hand, inhibition of autophagy has also been shown to enhance the effectiveness of anticancer therapies (9). Autophagy plays diverse roles in cancer, including protecting against cancer and contributing to the growth of cancer (10). The evidence supports the strong necessity of understanding the function of autophagy during disease development. Despite these research progresses, detailed information on autophagy–disease relationships are scattered in literature and there is a lack of online bioinformatics repository for these associations. In addition, although nowadays more and more autophagy-related genes and molecular regulatory pathway have been found (11, 12), the details of molecular mechanism about autophagy and human diseases are still not clear (13).

For the reason above, a resource of annotation system through which researchers can obtain autophagy–disease relationships and the related molecular information is needed. Therefore, we developed a manually curated database entitled ‘Autophagy To Disease’ (ATD, http://auto2disease.nwsuaflmz.com), which provides a comprehensive resource of the molecular mechanism about autophagy and disease. ATD aims to dig out the links between autophagy and human diseases and to provide hints to the cures of these diseases through the molecular pathway of autophagy.

Based on the big data from ATD, we found that some classes of disease tend to relate with autophagy, including respiratory disease, cancer, urogenital disease and digestive system disease, while some other diseases show distant relationship with autophagy, including developmental disease, ear, nose and throat diseases, hematological disease and dermatological disease. We also found that some classes of autophagy-related diseases (ADs) have a strong association among each other and constitute modules. Furthermore, we extracted the autophagy–disease-related genes (ADGs) from ATD and provided a novel algorithm Optimized Random Forest with Label model (ORFL) to predict potential ADGs.

## Materials and methods

### Data collection and web interface implementation

In order to get a robust disease information, we tried to integrate different resources into one entire dataset as input to search literature. Finally, the category of diseases was referenced as existed database Online Mendelian Inheritance in Man (OMIM), Disease Ontology (DO) and published articles (14–17); and at last, 22 classes of disease that contain a total of 6215 detailed diseases were obtained (Table 1). In order to remove the redundancy of the disease names, we merged these 6215 diseases into 2557 diseases. Based on PubMed, which is a comprehensive database of biomedical literature, we extracted 16 356 related papers by searching with keywords ‘autophagy’ using eSearch and eFetch with the Structured Query Language statement. Then, we used the 2557 diseases as a lexicon to scan the candidate literature. Finally, 5478 papers were extracted and 318 different kinds of diseases were hit in these papers. All of the above processes were achieved with Perl as well as E-Utilities, which was used as an interface to OMIM and PubMed. Furthermore, in order to reduce the false positive rate, we filtered the 5478 autophagy-related articles manually, and at last, 1264 literature were retained for further research. In the process, we retained the connection between autophagy and disease in papers. Besides, we required that there must be gene/protein information in the remained papers.

Based on the above dataset, our online database ATD was built by MySql + PHP + Apache structure. This website is user-friendly and provides the function of search, analysis and ADG prediction with our novel algorithm ORFL. Users can search the database with different types of keywords, for example, the name of disease, gene and their relationship. Users can also analyze the Gene Ontology function, KEGG pathway and the potential chemical drug of ADGs in our website. Besides, we provided the ADG prediction function with our algorithms ORFL.

**Table 1:** Statistics of different classes of diseases in original datasets, filtered datasets and the final results with ADs

Disease class	Original disease		Filtered disease		AD	
	Number	Rate	Number	Rate	Number	Rate
Cancer	627	10.09%	159	6.22%	65	17.20%
Cardiovascular	395	6.36%	110	4.30%	20	5.29%
Connective tissue	52	0.84%	30	1.17%	4	1.06%
Dermatological	338	5.44%	129	5.04%	13	3.44%
Developmental	409	6.58%	161	6.30%	7	1.85%
Digestive system	128	2.06%	40	1.56%	14	3.70%
Ear, nose, throat	81	1.30%	22	0.86%	2	0.53%
Endocrine	259	4.17%	85	3.32%	11	2.91%
Hematological	333	5.36%	162	6.34%	16	4.23%
Immunological	251	4.04%	122	4.77%	29	7.67%
Metabolic	503	8.09%	357	13.96%	40	10.58%
Multiple	741	11.92%	374	14.63%	28	7.41%
Muscular	263	4.23%	74	2.89%	21	5.56%
Neurological	603	9.70%	241	9.43%	46	12.17%
Nutritional	39	0.63%	8	0.31%	2	0.53%
Ophthalmological	459	7.39%	141	5.51%	15	3.97%
Psychiatric	126	2.03%	33	1.29%	4	1.06%
Renal	99	1.59%	50	1.96%	7	1.85%
Respiratory	52	0.84%	21	0.82%	9	2.38%
Skeletal	313	5.04%	153	5.98%	16	4.23%
Unclassified	134	2.16%	80	3.13%	7	1.85%
Urogenital disease	10	0.16%	5	0.20%	2	0.53%
Total	6215	100.00%	2557	100.00%	378	100.00%

### Analysis of ADs

In order to observe the relationships between autophagy and disease, we designed two measurements; the first one is concentration ratio (CR) and the other one is retention ratio (RR). CR can be used as an indicator of concentration tendency: if one disease is more likely to be related to autophagy compared with all disease as background values, then it will show high CR value. When CR of a disease is greater than one, it means that the disease is positively related to autophagy compared with background values. RR is used to measure the proportion of diseases that retain in selected papers from lexicon: the higher the RR, the more diseases are retained. The range of RR value is from zero to one. The formulas are as follows:

(1) \begin{align*} {\mathrm{CR}}_{\mathrm{i}}=\frac{\mathrm{P}\left(\frac{\mathrm{Number}\ \mathrm{of}\ \mathrm{disease}\ \mathrm{i}}{\mathrm{Number}\ \mathrm{of}\ \mathrm{all}\ \mathrm{diseases}}|\mathrm{papers}\right)}{\mathrm{P}\left(\frac{\mathrm{Number}\ \mathrm{of}\ \mathrm{disease}\ \mathrm{i}}{\mathrm{Number}\ \mathrm{of}\ \mathrm{all}\ \mathrm{diseases}}|\mathrm{lexicon}\right)} \end{align*}

(2) \begin{equation*} {\mathrm{RR}}_{\mathrm{i}}=\frac{\mathrm{N}\left(\mathrm{disease}\ \mathrm{i}|\ \mathrm{papers}\right)}{\mathrm{N}\left(\mathrm{disease}\ \mathrm{i}|\mathrm{lexicon}\right)} \end{equation*}

where i means a class of disease i, $\mathrm{P}\left(\frac{\mathrm{Number}\ \mathrm{of}\ \mathrm{disease}\ \mathrm{i}}{\mathrm{Number}\ \mathrm{of}\ \mathrm{all}\ \mathrm{diseases}}|\right. \mathrm{papers}\Big)$ means the number of disease i divided by the total number of diseases retained in papers and $\mathrm{N}\left(\mathrm{disease}\ \mathrm{i}|\ \mathrm{papers}\right)$ means the number of disease i in papers.

We then analyzed the relationships between different kinds of ADs. This is based on the hypothesis that two different ADs are rarely seen in the same literature, so if two ADs appear in the same literature, it may indicate that there are some common properties and a strong association between these two ADs. Then cumulative hypergeometric distribution test was performed with the following formulas:

(3) \begin{equation*} P=1-\sum\nolimits_{\mathrm{i}=0}^{\mathrm{n}-1}\frac{\left(\begin{array}{@{}c@{}}\mathrm{M}\\{}\mathrm{i}\end{array}\right)\left(\begin{array}{@{}c@{}}\mathrm{N}-\mathrm{M}\\{}\mathrm{n}-\mathrm{i}\end{array}\right)}{\left(\begin{array}{@{}c@{}}\mathrm{N}\\{}\mathrm{n}\end{array}\right)} \end{equation*}

where N means the total number of literature about disease a or b, n is the number of literature about disease a and b and M is the number of literature about disease a. All of the above analyses were performed using R with version 3.1.2.

### ADG prediction with ORFL

Based on the above collected literature, 61 genes related to ADs were extracted using text mining technology (Table 2). The gene names were filtered from the literature with MEDLINE format files using the gene/protein names recognition algorithm AbGene, Bethesda, Maryland (18). In order to reveal the function of these genes, Gene Ontology (http://geneontology.org) was applied as a platform for gene enrichment analysis in this study (19).

**Table 2:** 61 genes are identified as ADGs by text mining

AKT2	BECN1	DISC1	GBP6	MTM1	SLC6A14	WRN
ARID5B	BNIP3	DRAM1	GBP7	MTMR14	SOX1	
ATAD3A	BNIP3L	EI24	HDAC3	MTOR	TET3	
ATG12	CD80	EPG5	HDAC5	NOD2	ULK1	
ATG13	CDKN1B	EPHB2	HMGN5	OPA1	UVRAG	
ATG3	CEP55	FOXO1	KL	PINK1	VCP	
ATG5	CHMP2B	GABARAP	LMNA	PTEN	VMA21	
ATG7	CIC	GABARAPL1	LRRK2	RAB25	VMP1	
BAX	CISD2	GABARAPL3	MCL1	RB1CC1	WDR45	
BCL2	DEPTOR	GBP1	MEG3	RHBDF1	WIPI1	

To predict more genes related to autophagy and human diseases, an artificial intelligence algorithm based on random forest was created. This new algorithm was named ‘Optimized Random Forest with Label model’ (ORFL). This algorithm is a type of semi-supervised prediction algorithm. The 61 ADGs were used as positive set, while negative set was randomly selected from all other human genes. The number of random sampling was 100 000. The detailed procedure can be seen in Figure 4. In this prediction algorithm, both information of histone modifications and transcription factor binding sites were integrated to comprise the features. The transcription factor binding sites of human were obtained from the University of California, Santa Cruz, database (http://genome.ucsc.edu/). Then, we got the histone modifications from Gene Expression Omnibus (http://www.ncbi.nlm.nih.gov/geo/) with accession number GSE16256, which originated from the Human Reference Epigenome Mapping Project (20, 21). Then, the data was extracted using Site Identification from Short Sequence Reads (22).

### Function similarity analysis

To verify the accuracy of the predicted ADGs with ORFL, functional similarity analysis was made based on function similarity analysis. The function similarity was analyzed with Lin’s algorithm based on the Gene Ontology Consortium (19, 23). It was processed with R version 3.1.2, using the GOSim package (24).

**Figure 1:**
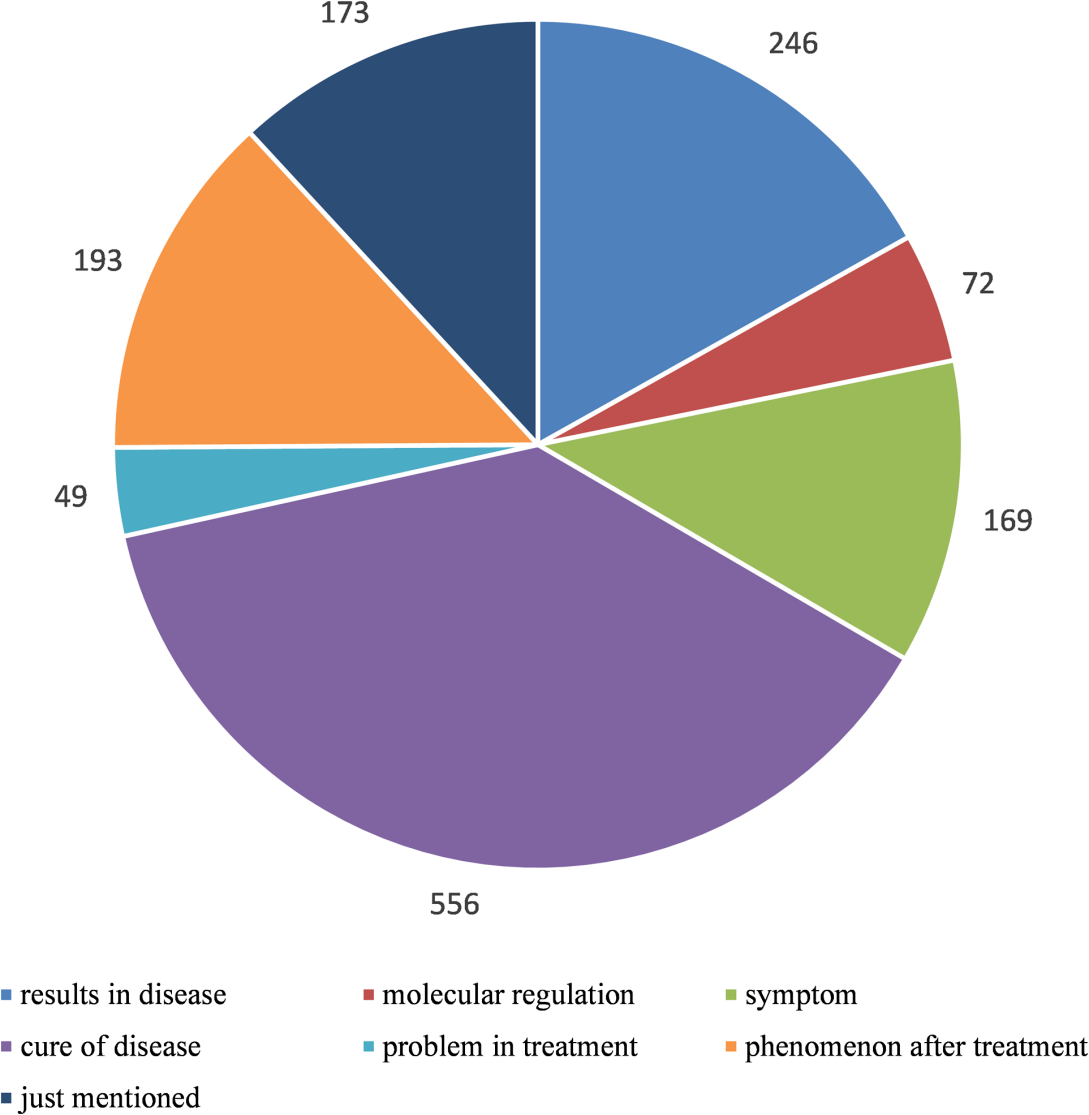
Statistics of relationships between autophagy and human diseases after manually curated annotation. We divided the relationships into seven types according to the degree of detail description in literature: ‘results in disease’ means autophagy presents as one of the results in disease, ‘molecular regulation’ means disease is caused by autophagy-mediated molecular regulation, ‘symptom’ means autophagy is a symptom as the disease, ‘cure of disease’ means the treatment of disease via autophagy pathway, ‘problem in treatment’ means autophagy is one of the problems during the treatment of disease, ‘phenomenon after treatment’ means autophagy presents as a phenomenon after treatment of disease and ‘just mentioned’ means there is no clear relationship between autophagy and disease and they are just present in the same literature.

## Results

### Manual curation of literature

In order to construct a high-quality database of curated AD, we used PubMed as a basis to search autophagy-related literature and collected genes, which were experimentally identified to be functional with AD. Finally, there were 5478 articles published before 2015. Only the genes with experimentally verified functions in AD are included in our database, and we call these genes as ADG. Furthermore, we focused on the relationship between autophagy and disease, so we filtered the 5478 autophagy-related articles with the disease information stored in OMIM and DO (14, 15), and at last, 1264 literature remained. By manual annotation with the 1264 papers that contain the information about disease and autophagy, seven main relations of human diseases and autophagy were found (Figure 1). During the manual annotation process, we filtered the literature without the information about the connection between autophagy and disease. Besides, there must be gene/protein information in the remained papers. It can be found that over 30% of articles are related to the cure of human diseases, and 19.46% of the passages show that autophagy gives rise to the human diseases.

### Association of different ADs

**Figure 2:**
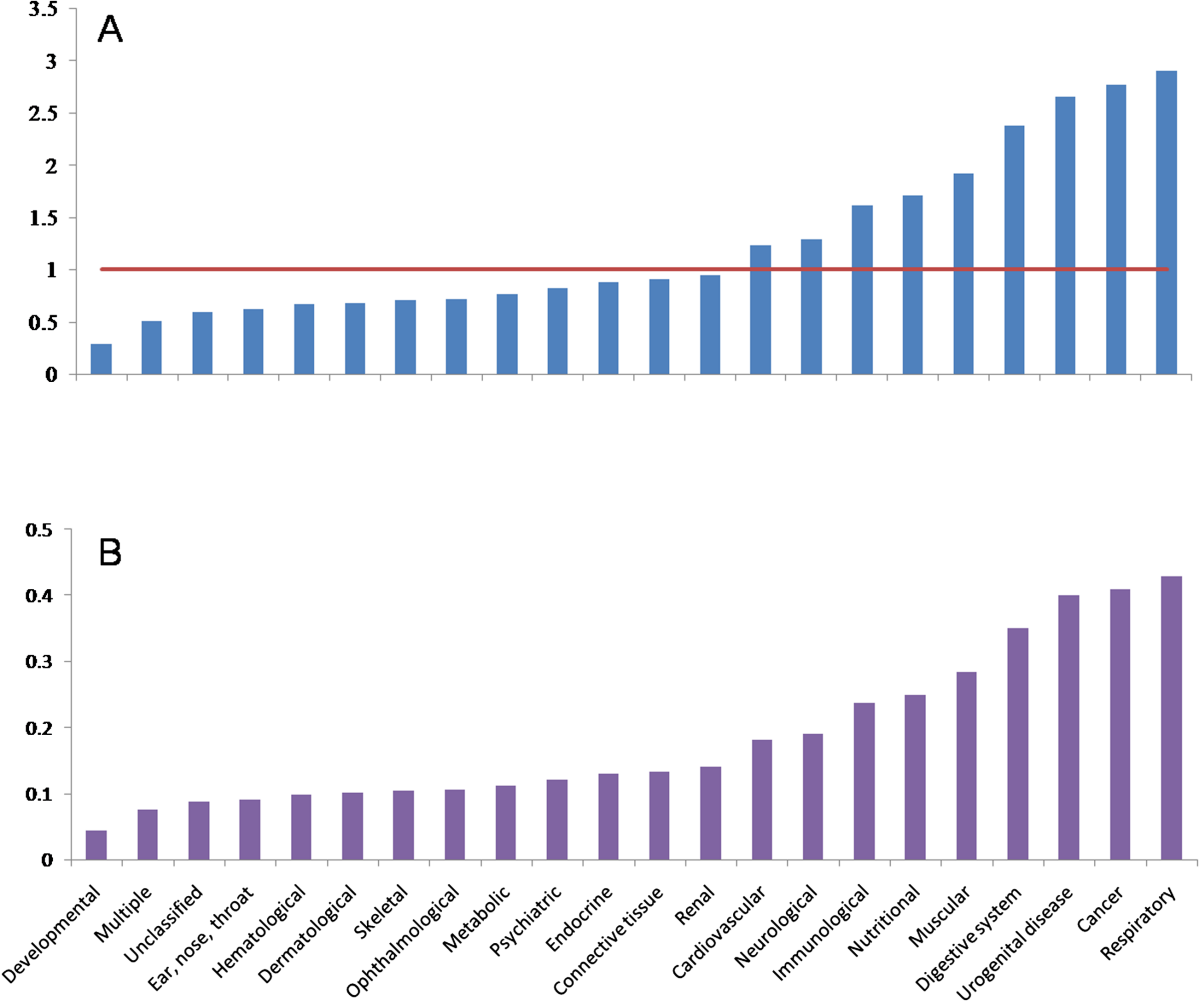
Distributions of CR and RR in different diseases. **A**, CR distribution. **B**, RR distribution.

There are 6215 diseases that were clarified in OMIM and DO primarily. In order to remove the redundancy of the diseases’ names, we merged these 6215 diseases into 2557 diseases, which are composed of 22 classes of diseases. After scanning the 5478 articles with the 2557 diseases as lexicon, 378 diseases and all 22 classes of disease remained. The composition of 22 classes of diseases has changed dramatically when the number of disease decreases from 2557 in lexicon to 378 in papers (Figure 2A). It can be found that the autophagy-related literature concentrates on some classes of diseases. In other words, CR of some classes of diseases increased, while other classes show opposite tendency. Respiratory, cancer, urogenital disease, digestive system disease, muscular disease, nutritional disease, immunological disease, neurological and cardiovascular disease show a higher CR, while developmental disease, ear, nose and throat diseases, hematological disease, dermatological disease, skeletal disease, ophthalmological disease, metabolic disease, psychiatric disease, endocrine disease, connective tissue disease, renal disease and multiple-type and unclassified diseases show a lower CR. We also found that some classes of diseases show higher RR when the number of disease decreases from 2257 to 378 (Figure 2B). Interestingly, it is also the same classes of diseases, which have higher CR, that show higher RR. This indicates that the composition of the final 378 diseases is affected by the selective pressure of autophagy.

**Figure 3:**
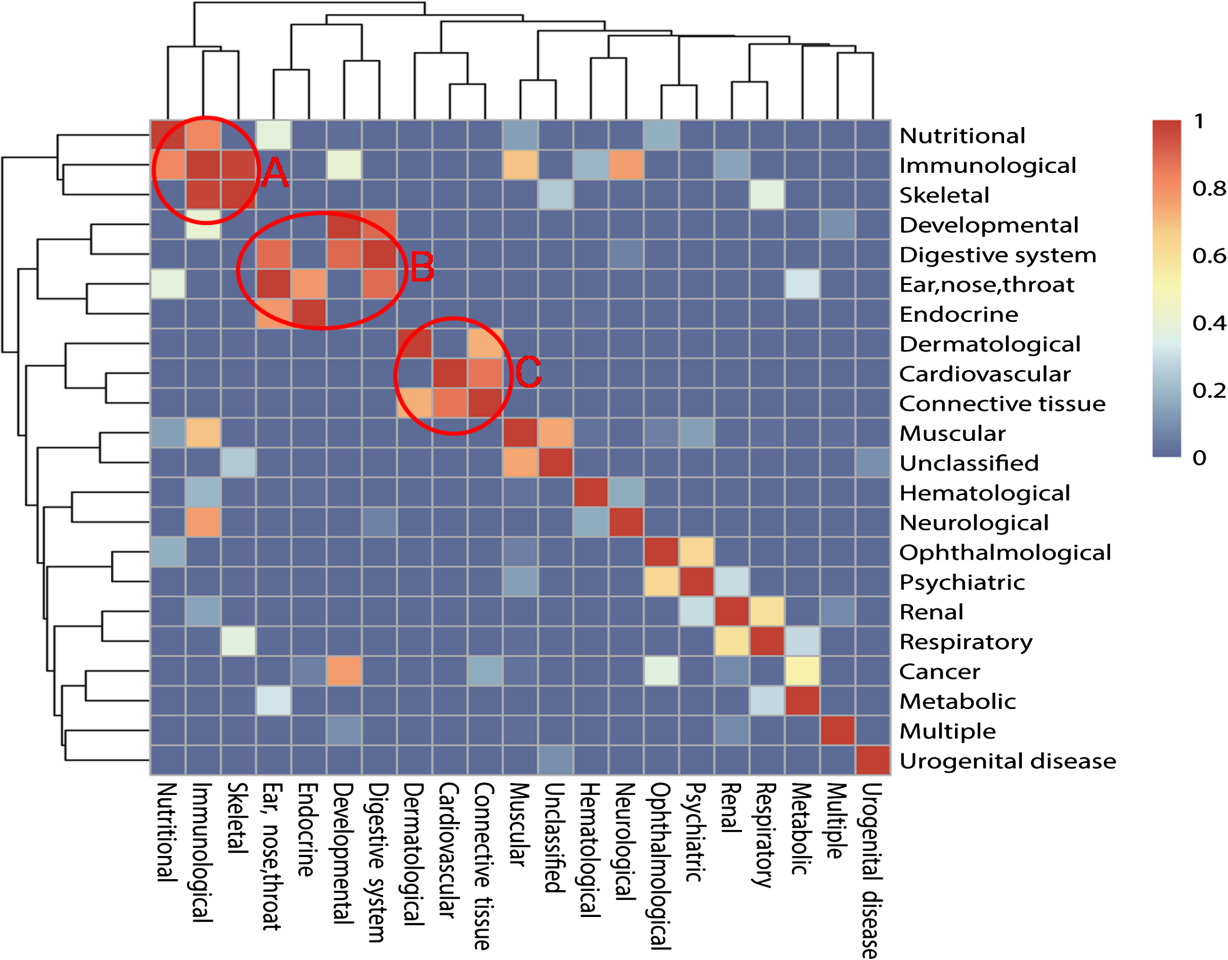
The relationship among 22 classes of diseases mediated with autophagy. The colors mean the significance values of hypergeometric distribution test; 1 means the highest value of significance, while 0 means the lowest value. There are three typical models after clustering: ‘A’ means Module 1, ‘B’ means Module 2 and ‘C’ means Module 3.

Considering the diversity of different classes of ADs, we tried to reveal the associations among them. We proposed a hypothesis that two different ADs are rarely seen in the same literature, so if two ADs appear in the same literature, it may indicate that there are some common properties and a strong association between these two ADs. Then, hypergeometric distribution test and cluster analysis were processed to verify the hypothesis. Results show that some classes of ADs have a strong association among each other and constitute modules. It can be found that there are some modules in the relationship map from Figure 3. Module 1 includes nutritional disease, immunological disease and skeletal disease. Module 2 includes developmental disease, digestive system disease, endocrine disease and ear, nose and throat diseases. Module 3 includes cardiovascular diseases, connective tissue diseases and dermatological diseases. Module 3 also includes ophthalmological diseases and psychiatric diseases. The diseases among the modules show a higher frequency to appear in the same literature than in background value with cumulative hypergeometric distribution test, which indicates the strong association between each other.

**Table 3:** Top 30 functions in Gene Ontology of ADGs.

Gene Ontology biological process complete	Bg gene count	ADGs count	*P*-value
Mitochondrial fragmentation involved in apoptotic process	9	3	0.0186
Nucleophagy	24	8	4.31E−11
Cellular response to nitrogen starvation	19	6	1.7E−07
Cellular response to nitrogen levels	19	6	1.7E−07
Negative regulation of cell size	10	3	0.0254
Mitochondrial outer membrane permeabilization	10	3	0.0254
Mitochondrion degradation	35	10	6.64E−14
Mitochondrial outer membrane permeabilization involved in programmed cell death	12	3	0.0438
Positive regulation of mitochondrial membrane permeability involved in apoptotic process	12	3	0.0438
Positive regulation of macroautophagy	17	4	0.00138
Autophagic vacuole assembly	44	10	6.43E−13
Organelle disassembly	49	10	1.87E−12
Mitochondrial fission	21	4	0.00319
Macroautophagy	59	11	1.45E−13
Positive regulation of response to nutrient levels	23	4	0.00457
Positive regulation of response to extracellular stimulus	23	4	0.00457
Negative regulation of mitochondrion organization	38	6	1.05E−05
Autophagy	141	21	5.59E−27
Regulation of mitochondrial membrane permeability	30	4	0.013
Negative regulation of autophagy	32	4	0.0168
Regulation of membrane permeability	33	4	0.019
Regulation of macroautophagy	42	5	0.00106
Regulation of release of cytochrome c from mitochondria	44	5	0.00134
Vacuole organization	102	11	5.47E−11
Cellular response to starvation	135	14	1.37E−14
Regulation of oxidative stress-induced cell death	39	4	0.0366
Neuron death	49	5	0.00226

Bg Gene Count and ADGs Count mean the background gene count and ADGs gene count annotated in the Gene Oncology Term

### Annotation of ADG

Among the 1264 articles with manual annotation, 61 genes were found to mediate autophagy and diseases (Table 2). In order to reveal the function of these genes, we processed the Gene Ontology enrichment and found that these genes were enriched on functions that include nuclear autophagy and mitochondrial fragmentation (Table 3).

**Figure 4:**
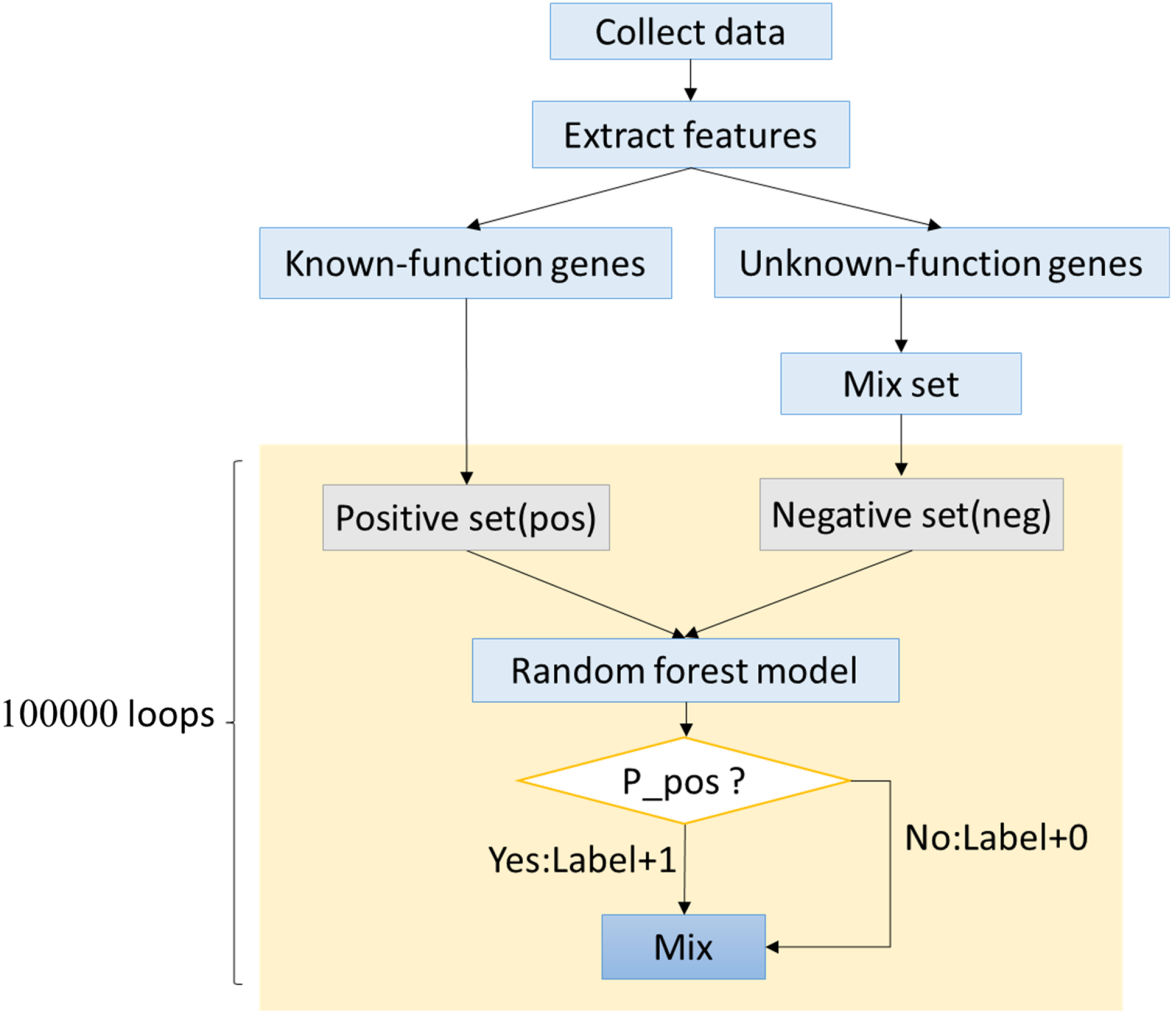
The framework of ORFL.

**Figure 5:**
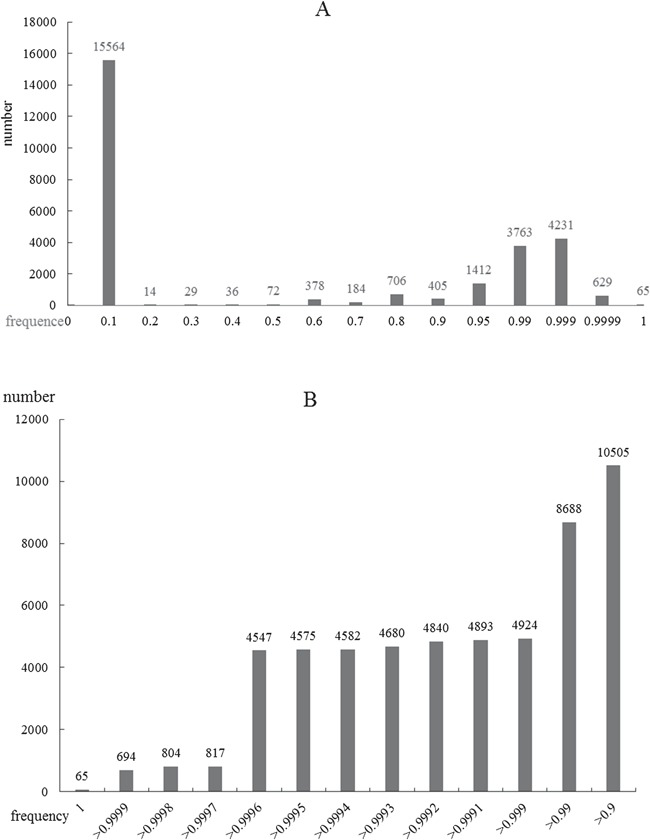
Frequency distributions of ORFL scores. **A**, Global distribution of scores from zero to one. **B**, Local precise distribution of scores from 0.9 to 1.

As an important physiological phenomenon, autophagy may involve complex pathways and a large number of genes. On the other side, the gene information that has been researched is destitute and is a bottleneck for the following molecular mechanism research. Considering the status, we proposed ORFL to predict genes that may be related to human diseases and autophagy (Figure 4). This model can provide a possibility score in prediction experiments, which includes 100 000 times of cycles. The possibility score of ADG relies on the frequency of prediction model; the higher the frequency in experiments, the higher the possibility scores. The panoramic distribution of the prediction results can be seen in Figure 5. It can be found that the total distribution obeys the law of bimodal distribution; one peak is on 0.1 and another one is on 0.999 (Figure 5A). This result indicates that there are two types of gene sets that have significantly different properties; the first one is irrelevant with an ADG and the other one is a potential ADG. In order to investigate the precise distribution of prediction results, we refined the distribution from 0.9 to 1 (Figure 5B). Two clear fault ages from the precise distribution result can be seen: the first one with 0.999 and the other one with 0.9997. For the facility of following ‘wet’ experiments, we provide the complete list of predicted ADGs with frequency as possibility score in Supplemental Materials (Table S1).

**Figure 6:**
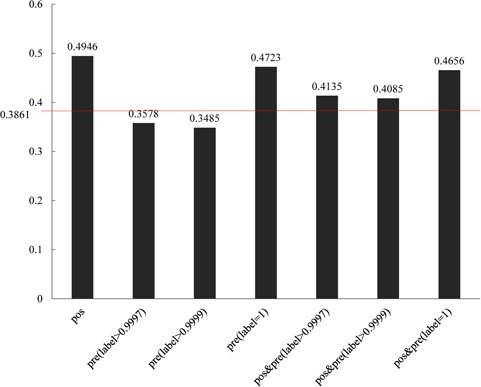
Comparisons of different groups of ADGs with gene functional similarity. 0.3861 is the background gene functional values of all human genes.

For the purpose of certificating the predicted ADGs, we processed a function consistency analysis based on Gene Ontology. The predicted ADGs were divided into three groups with thresholds of 0.999, 0.9997 and 1. Results show that all the three groups of predicted ADGs show similar functions with 61 positive ADGs (Figure 6). The inner similarities among predicted ADGs with a threshold of 1 also show higher function similarity consistency than the background value (0.3861). The other two groups of predicted ADGs show lower function similarity consistency than background value; the reason for this phenomenon is unknown.

## Discussion

Impaired autophagy has been observed in many injured tissues, and the failure of autophagy is thought to be one of the main reasons for the accumulation of cell damage and aging (4, 25). On the other hand, the atlas of relationships between autophagy and these diseases was missing. This study tried to provide the global perspective view about autophagy and diseases based on our manually curated database ATD. This database contains 5478 papers and 318 different kinds of diseases. As a comprehensive resource, ATD also provides the underlying gene information about autophagy and diseases. The gene information includes their involved function, pathway and chemical molecules. We believe that this will be helpful for the potential application of treatment for ADs.

Based on the above big data sets, we concluded that some classes of diseases, including cancer, metabolic disease, pulmonary disease, neurodegenerative disease, infectious diseases and vascular disease, show a close relationship with autophagy. Especially, we focused on the disease of cancer, which is occupied by nearly one-third of the total number of diseases. Research shows that during the cancer development, autophagy is activated primarily and then recovered to the normal level (26). During the development from early to advanced cancer, pathways that regulate the normal function of autophagy are impacted and then lead to lysosome dysfunction (27). Apart from this, it can be seen that neurodegenerative diseases also show a close relationship with autophagy. This is also consistent with a previous study. Nakai *et al.* found that in brain cells during starvation, autophagy may cause misfolded proteins accumulation, which may result in the damage of neurons and neurodegenerative diseases (28). The reason may be that specific misfolded proteins that expose the KFERQ degradation signal can be degraded by a branch of the autophagy–lysosome system (hereafter autophagy), in which substrates are directly delivered into lysosomes, leading to degradation by lysosomal hydrolases into amino acids (29). So on some stimulation, the pathway of autophagy will be dysfunctional and the degradation signal of misfolded proteins will be interrupted. More interestingly, through our website, we can find some connections between different diseases via autophagy. For example, when using ‘neuro’ as input or using ‘diabetes’ as input, we can both get the result from Towns’ work published in Autophagy (30), which provides a critical link between the immune system and the loss of function and eventual demise of neuronal tissue in type 2 diabetes.

We also detected the relationship among different classes of diseases and found some disease modules. Through phenotype connection, these disease modules reflect the shared molecular pathway among the modules. The relationships among different diseases were constructed using autophagy as mediation, which is similar to some previous works. Previous studies use drugs or genes as mediation (16, 17, 31).

In order to provide service about molecular mechanisms of ADs, this study predicted the genes related to ADs using our algorithm ORFL. This algorithm provided scores as an indicator to judge the probability of one gene to be related with ADs. The frame of ORFL is based on random forest and similar to our previous algorithm, the label method algorithm (32). Considering the up to 100 000 times of random sampling, we believe that this strategy will be robust. In fact, our function consistency analysis shows that ORFL is effective and can dig genes that have similar function with known positive ADGs. This function may be useful for the following research about the mechanisms of ADs, and we will pay more attention to the deep research in future.

In summary, this study provided a comprehensive annotation system about the relationship between autophagy and diseases. Comparing with some other databases related to the autophagy, it can be found that autophagy database (http://autophagy.info/autophagy/index.html) built in 2011 by Keiichi Homma *et al.* and the Human Autophagy Database are both focused on the autophagy in normal physiological condition (33, 34), while ATD focuses on the annotation of autophagy and human disease and link them with genes. Some other database, such as Autophagy Regulatory Network, provides molecular regulation relationship related with autophagy (35), but they only focus on normal physiological condition, without any disease information. Furthermore, compared with all of the above existed database, our ATD database provided potential novel ADGs using our algorithm ORFL, which may provide inspiration for the further cellular and molecular experiments. We believe that ATD is specific for the treatment of human disease through the molecular pathway of autophagy. In the future, there are several strategies to improve our database, including adding more literature, data resources, disease information resources, utilization of search engines and more ADGs prediction methods. These strategies will be helpful to increase the ADGs coverage and precision.

## Supplementary Material

Supplementary Data

## Notes

**Database URL:** http://auto2disease.nwsuaflmz.com
